# Unveiling the Role of Ecto-5′-Nucleotidase/CD73 in Astrocyte Migration by Using Pharmacological Tools

**DOI:** 10.3389/fphar.2018.00153

**Published:** 2018-03-01

**Authors:** Marija Adzic, Nadezda Nedeljkovic

**Affiliations:** ^1^Department of General Physiology and Biophysics, Institute for Physiology and Biochemistry, Faculty of Biology, University of Belgrade, Belgrade, Serbia; ^2^Centre for Laser Microscopy, Institute for Physiology and Biochemistry, Faculty of Biology, University of Belgrade, Belgrade, Serbia

**Keywords:** ecto-5′-nucleotidase/CD73, reactive astrocytes, cell adhesion, migration, scratch wound assay

## Abstract

CD73 is a bifunctional glycosylphosphatidylinositol (GPI)-anchored membrane protein which functions as ecto-5′-nucleotidase and a membrane receptor for extracellular matrix protein (ECM). A large body of evidence demonstrates a critical involvement of altered purine metabolism and particularly, increased expression of CD73 in a number of human disorders, including cancer and immunodeficiency. Massive up-regulation of CD73 was also found in reactive astrocytes in several experimental models of human neuropathologies. In all the pathological contexts studied so far, the increased expression of CD73 has been associated with the altered ability of cells to adhere and/or migrate. Thus, we hypothesized that increased expression of CD73 in reactive astrocytes has a role in the process of astrocyte adhesion and migration. In the present study, the involvement of CD73 in astrocyte migration was investigated in the scratch wound assay (SW), using primary astrocyte culture prepared from neonatal rat cortex. The cultures were treated with one of the following pharmacological inhibitors which preferentially target individual functions of CD73: (a) α,β-methylene ADP (APCP), which inhibits the catalytic activity of CD73 (b) polyclonal anti-CD73 antibodies, which bind to the internal epitope of CD73 molecule and mask their surface exposure and (c) small interfering CD73-RNA (siCD73), which silences the expression of CD73 gene. It was concluded that approaches that reduce surface expression of CD73 increase migration velocity and promote wound closure in the scratch wound assay, while inhibition of the enzyme activity by APCP induces redistribution of CD73 molecules at the cell surface, thus indirectly affecting cell adhesion and migration. Application of anti-CD73 antibodies induces a decrease in CD73 activity and membrane expression, through CD73 molecules shedding and their release to the culture media. In addition, all applied pharmacological inhibitors differentially affect other aspects of astrocyte function *in vitro*, including reduced cell proliferation, altered expression of adenosine receptors and increased expression of ERK1/2. Altogether these data imply that CD73 participates in cell adhesion/migration and transmits extracellular signals through interactions with ECM.

## Introduction

Ecto-5′-nucleotidase, known as CD73 (eNT; E.C. 3.1.3.5) is Zn^2+^-binding membrane enzyme with its active site facing the extracellular compartment (Zimmermann, 1992; Zimmermann et al., 2012). The enzyme is capable to dephosphorylate several ribo- and deoxyribonucleoside 5′-monophosphates to their corresponding nucleosides (Zimmermann et al., 2012), whereby 5′-adenosine monophosphate (AMP) is the most efficiently hydrolyzed substrate (Bianchi and Spychala, 2003). The enzyme has a broad tissue distribution, being expressed in many cell types, including subpopulations of T and B lymphocytes and in a number of tumor cells (Antonioli et al., 2013a). Thus, as the main source of extracellular adenosine in all tissues, it is of a major pharmacological interest.

The functional CD73 molecule comprises two identical subunits, tethered by non-covalent bonds and anchored to the outer leaflet of plasma membrane via glycosylphosphatidylinositol (GPI) anchor (Naito and Lowenstein, 1981; Ogata et al., 1990; Strater, 2006). Each enzyme monomer comprises 576 amino residues, organized in one flexible α-helical domain interposed between N- and C-terminal domains. Two 26-amino acid signal peptides in the N-terminal domains coordinate binding of two Zn^2+^ ions required for the catalytic activity, while two opposing C-terminal domains provide a binding site for AMP (Strater, 2006). Structure-function analysis revealed that a transition of the molecule between open and closed conformational states is required for the catalytic activity (Knofel and Strater, 2001; Knapp et al., 2012). The activity is competitively inhibited by adenosine diphosphate (ADP) (Cunha, 2001) or its analog, α,β-methylene ADP (APCP), which is the most potent CD73 inhibitor known to date. Mature CD73 contains four (human and mouse) or five (rat) *N*-glycosylation sites that can be completely or partially modified with a complex mixture of glycans (Zimmermann, 1992). As a result, in different tissues and cell types, CD73 occurs in several glycoforms, which differ in their apparent molecular weight (60–80 kDa) and sensitivity to lectins (Vogel et al., 1991; Zimmermann, 1992; Navarro et al., 1998). While bulk CD73 is membrane-bound glycoprotein attached to the cell surface via GPI anchor, the enzyme may be shed from the membrane by phosphatidylinositol-specific phospholipase or by proteolytic cleavage, to give rise to the soluble variant which retains its catalytic activity (Heuts et al., 2012). The soluble CD73 constitutes an important auxiliary system for maintaining extracellular nucleotide concentrations in blood and body fluids (Yegutkin, 2008; Heuts et al., 2012; Laketa et al., 2015).

CD73 has two major functions (Zimmermann et al., 2012). The first is a generation of extracellular adenosine from AMP, which derives from adenosine triphosphate (ATP) or nicotinamide dinucleotide (NAD+) released in the extracellular space. The nucleotides are crucially involved in cellular energy metabolism, but they also function as signaling molecules, after being secreted across cell membranes (Ziegler, 2000; Grahnert et al., 2011). In a response to diverse noxious stimuli in the brain, ATP and NAD+ are massively released out of cells, where they act as danger-associated molecular patterns (DAMPs) involved in an initiation of the immune reaction. Following their action at specific P2 purinoceptors (Abbracchio et al., 2006; Khakh and North, 2012), ATP and NAD+ are hydrolyzed to AMP by catalytic actions of ecto-nucleoside triphosphate diphosphohydrolase 1 (NTPDase1/CD39) (Zimmermann et al., 2012; Yegutkin, 2014) and nucleotide pyrophosphatase/phosphodiesterase 1 (NPP1/CD38) (Horenstein et al., 2013) respectively, thereby providing the substrate for CD73. Adenosine generated from AMP acts at G-protein coupled P1 purinoceptors, functionally linked to inhibition (A_1_, A_3_) or stimulation (A_2_A, A_2_B) of adenylate cyclase (Fredholm et al., 2001). Resulting adenosine is implicated in a broad range of physiological processes, including cell growth, differentiation and immune-suppression (Schwaninger et al., 1997; Hasko and Cronstein, 2004). In other words, extracellular pathways for a degradation of two danger signals, ATP and NAD+, converge toward CD73 and culminate in the formation of adenosine, which, in turn, exhibits strong tissue protective and anti-inflammatory actions (Antonioli et al., 2013b). Besides generation of adenosine, CD73 functions as a membrane receptor for extracellular matrix molecules (ECM), tenascin C, fibronectin and laminin (Stochaj et al., 1990; Olmo et al., 1992; Sadej et al., 2008). Interactions between cell adhesion molecules (CAM) and ECM play the key role in a regulation of cell adhesion, growth, migration, and differentiation, indicating that CD73 may participate in a control of these processes in both normal and neoplastic cells (Sadej et al., 2008).

Studies in a large number of human disorders demonstrate important role of CD73 in the immunity and cell communication (Schetinger et al., 2007; Deaglio and Robson, 2011; Ghiringhelli et al., 2012; Antonioli et al., 2013a,b; Gao et al., 2014) and highlight its potential as a pharmacologic target for immunomodulation and cancer treatment (Corbelini et al., 2015; Antonioli et al., 2016, 2017). Significant up-regulation of CD73 gene expression by reactive astrocytes was demonstrated in several experimental models of human neuropathologies, including ischemia (Braun et al., 1998), temporal lobe epilepsy (Bonan et al., 2000, Bonan, 2012), traumatic brain injury (Nedeljkovic et al., 2006; Bjelobaba et al., 2011), amyotrophic lateral sclerosis (Gandelman et al., 2010), experimental autoimmune encephalomyelitis (EAE) (Lavrnja et al., 2015) and glioma (Xu et al., 2013). In such conditions, astrocytes develop reactive phenotype, characterized by cellular hypertrophy and processes elongation (Sofroniew, 2009), they migrate to the area of tissue injury, where they interact with fibro-meningeal and NG2^+^ glial cells, release cytokines and deposit ECM to form a glial scar (Oberheim et al., 2008, 2012; Wiese et al., 2012; Wanner et al., 2013). Among many classes of molecules directly involved in different aspects of the altered cellular activity (Sofroniew, 2009; Wiese et al., 2012), reactive astrocytes massively increase the expression of CD73, both *in vivo* (Braun et al., 1998; Bonan et al., 2000; Nedeljkovic et al., 2006; Lavrnja et al., 2009, 2015; Gandelman et al., 2010; Bjelobaba et al., 2011; Bonan, 2012) and *in vitro* (Fenoglio et al., 1997; Bavaresco et al., 2008; Brisevac et al., 2012, 2015). The expression switch for CD73, however, does not turn on immediately, but in days following the initial tissue damage. Thus, in the model of stab brain injury *in vivo*, the contribution of CD73^+^ astrocytes to a total number of GFAP^+^ astrocytes gradually increases, reaching the maximum 14 days after the injury, when the patterns of CD73 and GFAP expression in reactive astrocytes completely overlap (Bjelobaba et al., 2011). The similar sustained pattern of CD73 expression is observed in EAE, where individual CD73^+^ astrocytes appear just at the peak of the symptomatic phase of the illness and their number continues to increase toward the end of the disease, in apparently recovered animals (Lavrnja et al., 2015). Thus, delayed expression of CD73 may be an important part of the complex molecular phenotype of reactive astrocytes in different neuropathologies. The local production of adenosine by CD73 and the consequent activation of A_1_R may account for numerous immunosuppressive actions of adenosine, such as reduced proliferation and enhanced protection of astrocytes from cell death (Ciccarelli et al., 1994, 2007; Tsutsui et al., 2004; Bjorklund et al., 2008).

Given that up-regulation of CD73 by reactive astrocytes represents a common phenomenon in neurological disorders associated with neuroinflammation, we suggest that CD73 might participate in a specific cellular activity performed by reactive astrocytes during course of reactive gliosis. Regarding the dual role of CD73 and its sustained up-regulation in neuroinflammatory conditions, we hypothesized that CD73 plays a role in the ability of reactive astrocytes to establish cell-ECM or cell–cell contacts that strengthen their ability to adhere to the substrate. Thus, we have applied diverse pharmacological tools to inhibit CD73 activity, to mask its surface exposure or to knock-down its membrane expression in cultured astrocytes, in order to study the involvement of CD73 in the cell migration in a scratch wound assay *in vitro*. We have found that approaches that reduce the number of CD73 molecules at the cell surface, either by application of anti-CD73 antibodies or CD73 gene knockdown, promote astrocytes migration *in vitro*. We have also demonstrated that application of anti-CD73 antibodies induces CD73 shedding from the cell membrane and activation of a downstream ERK1/2-mediated signaling, which implies physiological relevance of this interaction and a potential mechanism for CD73 regulation by its natural ligands *in vivo*.

## Materials and Methods

### Chemicals

Glucose, poly-L-lysine (PLL), Trypsin, EDTA, Triton^TM^ X-100, adenosine (Ado), adenosine 5′-triphosphate disodium salt hydrate (ATP), adenosine 5′-diphosphate sodium salt hydrate (ADP), adenosine 5′-monophosphate sodium salt hydrate (AMP), adenosine 5′-(α,β-methylene) diphosphate (APCP), bovine serum albumin (BSA), normal donkey serum (NDS), paraformaldehyde (PFA), protease inhibitor cocktail and Mowiol bedding medium were all purchased from Sigma–Aldrich (St. Louis, MO, United States). Leibovitz’s L-15 Medium, Penicillin/Streptomycin, Fetal Bovine serum (FBS), Dulbecco’s modified Eagles’s medium (DMEM), Opti-MEM (Reduced Serum Media) and Lipofectamine 2000 were obtained from Gibco. Small interfering RNA (siRNA) probes were purchased from Thermo Fisher Scientific (Carlsbad, CA, United States). 4,6-Diamidino-2-phenylindole (DAPI) was purchased from Molecular probes (Eugene, OR, United States). Immobilon Western Chemiluminescent HRP Substrate (Cat. #7632365) was obtained from Merck (KGaA, Darmstadt, Germany). Micro BCA Protein Assay Kit was purchased from (Thermo Fisher Scientific, Rockford, IL, United States).

### Animals

One to 2-day old rat male pups of Wistar strain from the local colony were used for primary cortical astrocyte culture preparation. The animal procedures were performed in compliance with European Communities Council Directive (2010/63/EU) and Serbian Laboratory Animal Science Association for the protection of animals used for experimental and the scientific purposes and were approved by the Ethics Committee of the Faculty of Biology, University of Belgrade. Authorization reference number EK-BF-2016/05.

### Primary Astrocyte Culture

Cerebral cortices were dissected and meninges were thoroughly removed in ice-cold phosphate–buffered saline (PBS). The cortices were mechanically dissociated by gentle pipetting under sterile conditions in Leibovitz’s L-15 isolating medium supplemented with 2 mM L-glutamine, 100 IU/ml penicillin, 0.1 mg/ml streptomycin, and 0.1% BSA. After two centrifuge/washing steps at 500 × *g* for 4 min, cell suspension was passed through ø 0.8 mm and ø 0.6 mm sterile needles, to remove residual tissue aggregates. Additional centrifugation step at 500 × *g* for 4 min was followed by cells resuspension in Dulbecco’s modified Eagle’s medium with the addition of 10% heat-inactivated FBS, 25 mmol/l glucose, 2 mmol/l L-glutamine, 1 mmol/l sodium pyruvate, 100 IU/ml penicillin and 100 μg/ml streptomycin. Cells were subsequently seeded in tissue culture flasks for adherent cells and grown at 37°C in a humified incubator with 5% CO_2_/95% air. Culture medium was replaced 1 day after the isolation and then every other day until cultures were 80–90% confluent. Primary microglia and oligodendrocytes were removed by vigorous shaking at 400 rpm for 16–20 h on a plate shaker (PerkinElmer, Turku, Finland) and additional mechanical washing using a 1-ml pipette if needed. Adherent primary astrocytes were washed with PBS, trypsinized (0.25% trypsin and 0.02% EDTA) and replated on new dishes at a density of 1.5 × 10^4^ cells/cm^2^ and maintained to reach confluence. Each cell culture was prepared from a single animal cortex. A total of 21 animals were used in the study.

### Treatments

After reaching the near confluence, cells were synchronized by shifting the serum concentration to 0.5% FBS for 24 h prior the experiment. A scratch wound was made in astrocyte monolayer, afterward the primary pharmacological treatments were applied. The following primary treatments dissolved in standard medium with 10% FBS were applied: (a) 100 μM APCP; (b) goat polyclonal anti-CD73 IgG (Santa Cruz Biotechnologies; # *v*-20; 1:500); (c) siCD73-RNA (50 nM). Dilutions of the pharmacological treatments were chosen in separate experiments, as those which block and/or inhibit CD73, without affecting astrocyte viability (**Supplementary Figure S1A**). ATP, ADP, AMP, and adenosine were applied at 1 mM concentration, separately or 30 min after the primary treatment (APCP or the anti-CD73 antibody). The 1 mM concentration of ATP was chosen based on its ability to induce up-regulation of CD73 (Brisevac et al., 2015) and strong activation of cortical astrocytes (Adzic et al., 2017) and given that the nucleotide is rapidly metabolized by ectonucleotidases to the downstream nucleotides and adenosine as a final product, the later were applied in the same concentration.

Stock solutions of ATP, ADP, AMP, adenosine (100 mM) and APCP (10 mM) were prepared in sterile water and kept at -20°C until use. The final concentration of the nucleotides was adjusted with normal medium.

### siCD73 Gene Silencing

CD73 gene knockdown was induced by small interfering RNA-mediated gene silencing. Cells (2 × 10^4^ cells/cm^2^) were grown on the 35-mm Petri dishes. After reaching near confluence cells were transfected with siRNA duplexes (Thermo Fisher Scientific). The optimum concentration of 50 nM siRNA was determined in the separate dose-response experiment (**Supplementary Figure S1B**). For each siRNA probe, 50 nM siRNA and 5 μl Lipofectamine 2000 were dissolved in 150 μl Opti-MEM in separate tubes, incubated 5–10 min at room temperature (RT) and after mixing the two components, left for another 20 min. Aliquot of 300 μl of the complex was added to each Petri dish. Transfection was carried out for 8 h in 10% FBS supplemented DMEM (without Penicillin/Streptomycin). The medium was changed to standard growing medium (complete DMEM) and cells were left in standard conditions up to 48 h to increase the efficiency of transfection. The scratch wound assay and the treatments were performed 48 h after the transfection, isolation of total RNA was performed after additional 4 h, while all other tests and analysis were performed 72 h after the transfection.

The efficiency of transfection was validated by performing a positive and a negative control of transfection. The cells transfected with a siRNA sequence corresponding to the coding region of GADPH gene served as a positive control of transfection, while cells transfected with non-specific siRNA duplex which did not affect any target gene was used as a negative control of transfection (siCTR). The cells were processed 48 and 72 h after the transfection to determine the expression of the target genes at the mRNA and protein levels, whereas the normalized values obtained in siCTR were defined as 100% and used as a reference. In cells transfected with siGAPDH, the expression of GAPDH gene was considerably silenced at mRNA (10.3%) and protein levels (12.1%) with respect to siCTR. In culture transfected with siCD73, the expression of GAPDH gene was unaffected, whereas the expression of CD73, at mRNA and the protein levels, was reduced to about 51 and 36% relative to control, respectively.

### Scratch Wound Assay

Astrocytes were seeded at a density of 2 × 10^4^ cells/cm^2^ on the 35-mm Petri dishes for adherent cells and were maintained until reaching near confluence. Wound healing assay was performed by the method of Kornyei et al. (2000), as previously described (Adzic et al., 2017). A wound was made in astrocyte monolayer, by scraping the bottom of the dish with a sterile 200-μl pipette tip. Three to four scratches were made per each Petri dish in a defined geometry (**Supplementary Table S1**). The treatment was applied to the cultures immediately after the wound was made and the cultures were further maintained in normal growth medium. Up to 16 random fields per each dish were captured at 0 h time on Carl Zeiss AxioObserver A1 inverted microscope (A-Plan 10× objective) by EM512 CCD Digital Camera System (Evolve, Photometrics). Consecutive images of selected microscopic fields were then captured at the time points of interest (4, 8, 12, 24, and 48 h) and stored as digitalized data. Wound area (μm^2^) and wound width (μm) were determined using ImageJ software package for each frame and time point. The wound closure (%) under the control and treatment conditions was assessed by expressing the closed wound area at each time point as a percentage of the initial wound area (0 h). Data are expressed as mean closed area (% ± SEM), from *n* ≥ 7 separate culture preparations. The values of mean closed wound area were plotted as a function of time and fitted to the logistic growth curve (Origin 8.0) to generate kinetic parameters, maximum closure velocity *V*_max_ (% closure/h) and slope of linear growth phase (s, %/h). The slope of the linear growth phase (%/h) was used to calculate cell front displacement (μm/h).

### Immunofluorescence and Confocal Microscopy

Astrocyte grown on glass coverslips (15 mm) were subjected to SW and treated as described. Twenty-four hours after the treatment, cells were prefixed in 4% PFA and blocked (1 h, RT) with a solution containing 10% NDS and 2% BSA in 0.01 M PBS. After the overnight incubation at +4°C with primary rabbit anti-rat CD73 antibodies (in 1% NDS and 1% BSA in 0.01 M PBS), cells were washed and incubated with secondary donkey anti-rabbit Cy3 IgG antibodies (2 h, RT). Cells were permeabilized with 0.01% Triton X-100 (15 min, RT) and subjected to the same blocking procedure. In the following round, cells were incubated with mouse anti-rat GFAP antibodies (1 h, RT) and secondary donkey anti-mouse Cy2 IgG antibodies (2 h, RT). Finally, nuclei were counterstained with DAPI (10 min, RT) and the cells were mounted on microscope slides with Mowiol solution. Incubation with the appropriate rabbit pre-immune serum instead of the primary anti-CD73 antibody resulted in the absence of any specific reaction.

Images of microscopic fields were captured with the confocal laser-scanning microscope (LSM 510, Carl Zeiss GmbH, Jena, Germany) using Ar Multi-line (457, 478, 488, and 514 nm) and HeNe (543 nm) lasers using 63× DIC oil objective and monochrome camera AxioCam ICm1camera (Carl Zeiss GmbH, Germany). Images were quantified using ImageJ software by calculating corrected total cell fluorescence (CTCF) for each frame using the following formula: CTCF = Integrated Density - (Area of selected cell × Mean fluorescence of background readings). The results present mean integrated fluorescence density ± SEM, from 15 frames for each treatment (*n* = 3 separate culture preparations, 5 frames per coverslip).

For Ki67/DAPI fluorescence staining, cells were fixed and permeabilized with 0.05% Triton X-100 and then blocked in 5% BSA in 0.01M PBS. Primary rabbit anti-Ki67 antibodies were applied overnight in 2% BSA at +4°C, followed by the incubation with the secondary donkey anti-rabbit Cy3 antibody for 2 h, on RT. The total nuclei were stained with DAPI (1:4000, for 10 min, on RT). Coverslips were mounted on microscopic slides with Mowiol solution. Images of microscopic fields were captured with Carl Zeiss Axio Observer A1 inverted epifluorescence microscope (A-Plan 10× objective) by EM512 CCD Digital Camera System (Evolve, Photometrics). Incubation without primary antibodies resulted in the absence of a specific reaction. The results are expressed as the percent of Ki67^+^ cells in total cell number (DAPI), determined from *n* = 2 separate culture preparations, and 5–7 frames/coverslip.

### Isolation of Cell Lysates and Separation of Culture Media

Astrocytes (2 × 10^4^ cells/cm^2^) were seeded on 60-mm diameter Petri dishes. After reaching confluence, cells were subjected to SW and treated as described. For the isolation of total proteins, cells were collected using warm 0.01 M PBS, centrifuged for 5 min at 500 × *g* and then re-suspended in 500 μl of cold RIPA lysis buffer, supplemented with 0.5% w/v protease inhibitor cocktail. The suspension was kept on ice for 30 min and subsequently centrifuged at 10000 × *g* for 10 min, at 4°C (Beckman, JA-20). The supernatant was carefully separated from the pellet, and the protein concentration was determined using BCA protein assay kit, according to manufacturer’s instruction. Culture media were removed and centrifuged for 10 min at 500 × *g* (Beckman, TA-10) to pellet residual cells. The supernatant was collected and used detection of soluble CD73 by dot blot procedure.

### Western Blot and Dot Blot Analysis

Samples were diluted in the 6× Laemmli sample buffer [375 mM Tris-HCl, pH 6.8, 12% sodium dodecyl sulfate (SDS), 60% (w/v) glycerol, 0.03% bromophenol blue], and the proteins prepared under reducing conditions were resolved on 4/12% SDS-PAGE gels. Proteins were electrotransferred to a PVDF support membrane (Immobilon-P transfer membrane, Millipore) and the membranes were blocked with 5% BSA in Tris buffer saline/Tween 20 (TBST). The overnight incubation with primary rabbit anti-rat CD73 antibodies (at 4°C), was followed by 2-h incubation with the appropriate secondary HRP-conjugated antibody. Support membranes were washed several times in TBST, and the bands were visualized with the use of ECL solution (Immobilon Western Chemiluminescent HRP Substrate, #7632365, Millipore) on a Chemi Doc-It imaging system (UVP, Upland, CA, United States).

The presence of soluble CD73 in culture media was detected by spotting 300-μl aliquots of culture media on PVDF support membrane (Immobilon-P transfer membrane, Millipore), through a vacuum-based Minifold dot blot apparatus (Schleicher & Schuell Inc., Keene, N.H.). The support membrane was blocked with 5% BSA in TBST and probed either with secondary donkey anti-goat HRP-conjugated IgG antibodies, or by a set of primary rabbit anti-rat CD73 and donkey anti-rabbit HRP conjugated IgG, followed by a visualization procedure, using ECL solution. Blots were washed in TBST and the chemiluminescent signal was detected on Chemi Doc-It imaging system (UVP, Upland, CA, United States). Media collected from *n* = 4 separate cultures were used in the analysis.

A list of used primary and secondary antibodies used for immunofluorescence, Western blotting, and dot blotting procedure is given in **Table 1**.

**Table 1:** List of primary and secondary antibodies.

Antibody selectivity	Source and clonality	Dilution and application	Manufacturer	RRID
CD73	Goat, *pc*	1:500, T	Santa Cruz (V-20), sc-14682	AB_2154099
CD73	Rabbit, *mc*	1:1500, WB	Cell Signaling (D7F9A), #13160	AB_2716625
CD73	Rabbit, *pc*	1:100, IF	ectonucleotidases-ab.com, Cat# [rNu-9L(I_4_,I_5_)]	
GFAP	Mouse, *mc*	1:200 IF	Sigma–Aldrich, G-3893	AB_477010
GFAP	Rabbit, *pc*	1:10000 WB	DAKO, Z0334	AB_10013382
Ki67	Rabbit, *pc*	1:500, IF	Abcam, ab15580	AB_443209
p44/42 MAPK	Rabbit, *mc*	1:1000, WB	Cell Signaling, #4695	AB_390779
GAPDH	Goat, *pc*	1:1000, WB	Santa Cruz (V-18), sc-20357	AB_641107
Anti-goat HRP-conjugated IgG	Donkey, *pc*	1:10000, WB, DB	Santa Cruz, sc-2020	AB_631728
Anti-rabbit HRP-conjugated IgG	Donkey, *pc*	1:10000, WB, DB	Santa Cruz, sc-2305	AB_641180
Anti-rabbit IgG Cy3	Donkey, *pc*	1:500 IF	Jackson ImmunoResearch, 711-165-152	AB_2307443
Anti-mouse IgG Cy2	Donkey, *pc*	1:500 IF	Jackson ImmunoResearch, 715-225-151	AB_2340827

*T, anti-CD73 blockade; WB, western blot; DB, dot blot; IF, immunofluorescence; pc, polyclonal; mc, monoclonal.*

### Quantitative Real-Time PCR

Astrocytes were seeded in 6-well plates at 2 × 10^4^ cells/cm^2^ density. After reaching near confluence (∼90%), the cultures were subjected to SW and treated as described. Four hours after the treatments, total RNA was extracted in TRIzol and RNA concentration and the purity were determined by measuring the absorbance at 260 nm and 260 nm/280 nm and 260 nm/230 nm ratios, respectively. A volume equivalent of 1 μg of total RNA was used to generate cDNA (High Capacity cDNA Reverse Transcription Kit, Applied Biosystems, Foster City, CA, United States), used for real-time PCR analysis (QuantStudio^TM^ 3 Real-Time PCR System, Applied Biosystems, Foster City, CA, United States). The reaction mixture contained 2 μl cDNA (10 ng/μl), 5 μl QTM SYBR Green PCR Master Mix (Applied Biosystems, Foster City, CA, United States), 0.5 μl primers (100 pmol/μl) and 2 μl RNase-free water (UltraPure, Invitrogen, Germany). Amplification was carried out under the following conditions: 10 min of enzyme activation at 95°C, 40 cycles of 15 s denaturation at 95°C, 30 s annealing at 64°C, 30 s amplification at 72°C and 5 s fluorescence measurements at 72°C. Relative target gene expression was calculated by the 2^-Δ^*^C^*^t^ method, using GAPDH gene as internal gene control. In transfection studies, actin was used as the internal control. Melting curves and gel electrophoresis of the PCR products were routinely performed to determine this specificity of the PCR reaction (not shown). Primer sequences are listed in **Table 2**. Results present mean target gene expression (relative to GAPDH) ± SEM, from *n* separate determinations performed in duplicate.

**Table 2:** List of primer pairs for rtPCR.

Target gene	Forward	Reverse
*Nt5e/CD73*	CAAATCTGCCTCTGGAAAGC	ACCTTCCAGAAGGACCCTGT
*Adora1*	GTGATTTGGGCTGTGAAGGT	GAGCTCTGGGTGAGGATGAG
*Adora2a*	TGCAGAACGTCACCAACTTC	CAAAACAGGCGAAGAAGAGG
*Adora2b*	CGTCCCGCTCAGGTATAAAG	CCAGGAAAGGAGTCAGTCCA
*Adora3*	TTCTTGTTTGCCTTGTGCTG	AGGGTTCATCATGGAGTTCG
*GAPDH*	TGGACCTCATGGCCTACAT	GGATGGAATTGTGAGGGAGA
*HPRT*	GGTCCATTCCTATGACTGTAG	CAATCAAGACGTTCTTTCCAGTT

### 5′-AMP Phosphohydrolase Assay and Free Phosphate Determination

Astrocytes were seeded on 24-well plate, at a density of 6 × 10^4^ cells per well. After reaching near confluence, cultures were subjected to SW and treated as described. Cells were washed 3 × 5 min with phosphate-free medium (117 mM NaCl, 5.3 mM KCl, 1.8 mM MgCl_2_, 26 mM NaHCO_3_, 10 mM glucose, pH 7.4) to eliminate cell debris and free phosphates. 5′-AMP phosphohydrolase activity was assayed by determining free phosphates, liberated as a result of the enzyme reaction. The reaction was initiated by adding 240 μl of 1 mM AMP (in phosphate-free medium) to each well and the plates were incubated at 37°C for 30 min. The reaction was stopped by transferring the reaction volume to tubes already containing 24 μl ice-cold 3 mM PCA. Enzymatic hydrolysis of AMP and a level of inorganic phosphates liberated as a result of the enzymatic reaction were determined by the malachite green assay method, using KH_2_PO_4_ as a standard. The content of free phosphates liberated by non-enzymatic hydrolysis of AMP was corrected by assaying the activity in the reaction mixture without AMP and adding the substrate to the reaction volume after the addition of PCA. A contribution of tissue non-specific alkaline phosphatase (TNAP) to 5′-AMP phosphohydrolase activity was determined by assaying 5′-AMP hydrolysis in the presence of TNAP inhibitor levamisole (100–300 μM). Total protein content was determined by adding 100 μl of RIPA buffer to each well and using Micro BCA Protein Assay Kit (Thermo Fisher Scientific, Rockford, IL, United States), according to manufacturer instructions.

Free phosphate concentration was detected by a malachite green assay (Baykov et al., 1988). Aliquots (20 μl) of a working solution (0.1% of malachite green in 20% H_2_SO_4_, 7.5% ammonium molybdate and 11% Tween 20 in a ratio 10:2.5:0.2) were added to 80-μl aliquots of the reaction mixtures. The absorbance was measured at 620 nm and the amount free phosphates (Pi) liberates as a result of enzyme reaction was corrected for the non-enzymatic hydrolysis and presented as mean specific activity (nmol Pi/mg/min) ± SEM, from *n* separate culture preparations, performed in sextuplicate.

### Data Analysis

Raw data from the scratch wound assay were plotted as a function of time and fitted to the logistic growth curve using OriginPro8 SR0 software package (v8.0724, OriginLab Corporation, Northampton, MA, United States). Parameters of migration (maximum wound closure and slope of linear growth phase were derived as a logistic growth curve parameters. Comparison between means was performed by one-way ANOVA, followed by Tukey’s *post hoc* test and the differences were considered significant at the level of *p* < 0.05). Data are presented as mean ± SEM (from *n* separate cell culture preparations).

## Results

### Characterization of Primary Culture System

**FIGURE 1:**
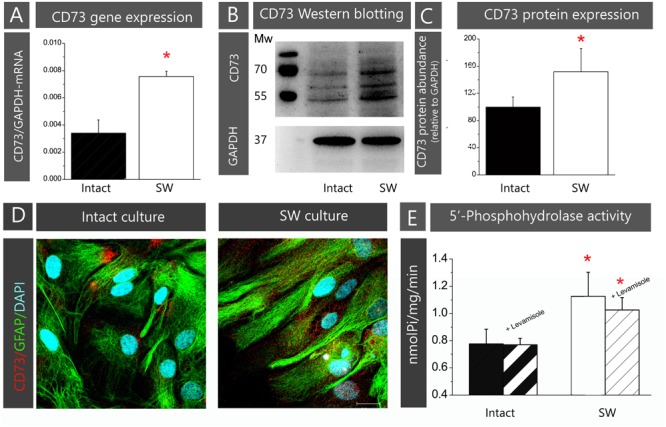
Validation of primary astrocytes culture model. The contents of CD73 in astrocyte cultures prior to (intact) or after creating the scratch wound (SW) were analyzed at mRNA, protein and functional level. **(A)** Expression of CD73 gene was determined by rt-PCR. Bars represent mean ± SEM of CD73-mRNA abundance relative to GAPDH, determined in *n* = 3 separate intact (*black bar*) and SW (*white bar*) culture preparation. **(B)** The abundance of CD73 protein was analyzed by Western blotting, after resolving whole cell lysates proteins on SDS-PAGE and probing the support membrane with the anti-CD73 antibodies (1:1500 in TBST; Cell Signaling, US). The antibodies recognized three protein bands on blots, with molecular weights of ∼69, 61, and 55 kDa. **(C)** Relative abundance of CD73 protein in each sample was assessed by measuring combined optical density of all bands in each lane by using ImageJ and by expressing the value relative to the optical density of GAPDH band in the same lane. The value obtained for intact culture was arbitrarily defined as 100% and was used as a reference (*black bar*). The bars represent mean CD73 relative protein abundance (% ± SEM) from *n* = 3 separate culture preparations. **(D)** Representative confocal images showing cell morphology and membrane topography of CD73 in confluent astrocyte monolayer (*left*) and cultures subjected to scratch wound (*right*). The images were obtained by double immunofluorescence labeling for CD73 (*red fluorescence*) and GFAP (*green fluorescence*) and nuclear counterstaining with DAPI (*blue fluorescence*). Scale bar = 25 μm. **(E)** The level of 5′-AMP phosphohydrolase activity in whole cell culture (*black bars*) and SW (*white bars*) cultures, determined in assay conditions as described in section “Materials and Methods.” Levamisole (100 μM) was used as the alkaline phosphatase inhibitor (*striped bars*). The bars represent mean activity (nmol Pi/mg/min) ± SEM, from *n* = 3 separate culture preparations performed in sextuplicate. Significance inside the graphs: ^∗^ denotes significance at *p* < 0.05 in respect to intact culture.

In the present study, the role of CD73 in astrocyte adhesion and migration was investigated in the scratch wound assay using primary astrocyte culture prepared from neonatal rat cortex. The validity of the chosen cell model has been demonstrated by assessing the expression and function of CD73 in a confluent astrocytes monolayer, prior to (Intact) and after creating the scratch wound (SW). To avoid variations in CD73 levels due to differences in cell density, cell cycle, and culturing conditions, the measurements have been always done under the same conditions, described in section “Materials and Methods.” The cells in intact astrocyte monolayer expressed 5′-AMP phosphohydrolyzing activity at the level comparable to other rat primary astrocyte cultures (Bavaresco et al., 2008; Brisevac et al., 2012). After creating the wound, the abundance of CD73-mRNA increased more than twofold (223.3 ± 5.1%, *p* < 0.01; **Figure 1A**) in SW when compared to intact culture. The increase in CD73 gene expression was accompanied by an increase in total CD73 protein abundance (**Figures 1B,C**) and 5′-AMP phosphohydrolase activity (**Figure 1E**). Three protein bands were seen on Western blots, corresponding to glycosylated (∼69 and 61 kDa) and non-glycosylated (∼55 kDa) protein forms, with a combined abundance significantly higher in SW (151.6 ± 34.2%, *p* < 0.05) than in intact culture. Surface expression of CD73 was visualized by double CD73/GFAP-immunofluorescence labeling (**Figure 1D**). The cells in confluent monolayer displayed usual morphology *in vitro*, characterized by polygonal cell bodies with inconspicuous processes. The immunoreaction (*ir*) corresponding to CD73 is found in clusters at the corners of the polygonal cell bodies. In SW, the cells lining the wound edge developed protoplasmic processes extending to the wound area. These processes exhibited stronger GFAP-immunoreactivity, while fine punctuate CD73-*ir* was distributed all around the cells. Finally, the level of 5′-AMP phosphohydrolase activity was significantly higher in SW culture (1.13 ± 0.18 nmol Pi/mg/min, *p* < 0.05) than in intact culture (0.78 ± 0.11 nmol Pi/mg/min). The 5′-AMP hydrolyzing activities in intact and SW cultures were insensitive to alkaline phosphatase inhibitor levamisole, indicating that the activity belonged to CD73 (**Figure 1E**).

To confirm that the injury-induced upregulation of CD73 is associated with the reactive phenotype of cultured astrocytes, intact cells were activated by applying inflammatory mediators bacterial endotoxin LPS (100 ng/ml) or proinflammatory cytokine IL-1β (10 ng/ml) and the expression of CD73 was measured by immunofluorescence and by 5′-AMP phosphohydrolase assay after 24 h. Both proinflammatory mediators induced significant increase in surface expression of CD73 and upregulation of 5′-AMP phosphohydrolase activity in respect to non-treated control cells (**Supplementary Figure S2**).

### Reduced Cell Surface Expression of CD73 Promotes Wound Closure in Primary Astrocyte Culture

The scratch wound assay was used to study astrocyte migration *in vitro*. The images were captured immediately after creating the wound (0 h) and at 4-h intervals during 48 h (**Supplementary Figure S3A**) and the remaining wound area at each time point was measured and expressed as a percentage of initial wound area (at 0 h). The values were plotted vs. time and the curve was used to calculate the slope of linear growth phase (**Supplementary Figure S3B**). The percentage of the initial wound area covered between two consecutive time-points was used to calculate the velocity of wound closure (%/h), whereas cell front displacement was calculated as a displacement of the leading edge during 24 h (μm/h) (**Supplementary Figure S3C**).

To assess the role of CD73 in astrocyte migration, cell cultures were treated with one of the following pharmacological inhibitors of CD73: (1) anti-CD73 antibodies (goat polyclonal IgG, Santa Cruz Biotechnologies; # *v*-20; 1:500 in full growing medium), with epitope mapping within the internal region of CD73 molecule; (2) non-hydrolyzable ADP analog (100 μM α,β methylene ADP – APCP), which binds to the active site and inhibits CD73 catalytic activity, and (3) CD73 small interfering RNA (siCD73), which induces CD73 gene knockdown by neutralizing CD73-mRNA. The parameters of cell motility obtained in the scratch wound assays in SW conditions and in the presence of the inhibitors are given in **Table 3**.

**Table 3:** Cell motility parameters.

	*V*_max_ (% coverage/h)	Slope of linear growth phase (% coverage/h)	Cell front displacement velocity (μm/h)
Control	3.43 ± 0.52	3.11 ± 0.19	12.73 ± 0.77
Anti-CD73 Ab	4.56 ± 0.35^∗^	4.31 ± 0.20^∗^	17.66 ± 0.80^∗^
Anti-CD73 Ab + Ado	4.44 ± 0.48	3.88 ± 0.26	15.89 ± 0.04
Anti-CD73 Ab + AMP	4.04 ± 0.40	3.73 ± 0.45	14.05 ± 0.84
Anti-CD73 Ab + ATP	3.96 ± 0.28	3.83 ± 0.18^∗^	15.69 ± 0.75^∗^
α,β-methylene ADP	3.27 ± 0.37	2.94 ± 0.26	12.04 ± 1.06
α,β-methylene ADP + Ado	3.34 ± 0.28	2.72 ± 0.25	11.40 ± 1.01
α,β-methylene ADP + AMP	3.71 ± 0.14	3.29 ± 0.14	13.48 ± 0.57
α,β-methylene ADP + ATP	3.82 ± 0.61	2.58 ± 0.24	11.57 ± 0.98
Ado	2.92 ± 0.21^∗^	2.68 ± 0.17^∗^	10.99 ± 0.68^∗^
AMP	2.25 ± 0.08	1.98 ± 0.41^∗^	8.11 ± 1.68^∗^
ADP	1.99 ± 0.41^∗^	2.18 ± 0.23^∗^	8.93 ± 0.94^∗^
ATP	3.14 ± 0.25	3.74 ± 0.18^∗^	15.33 ± 0.76^∗^
siCTR	1.61 ± 0.27	1.63 ± 0.20	6.68 ± 0.82
siCD73	3.31 ± 0.62^∗^	2.34 ± 0.06^∗^	9.58 ± 0.24^∗^

*^∗^Significance at *p* < 0.05 in respect to control SW.*

**FIGURE 2:**
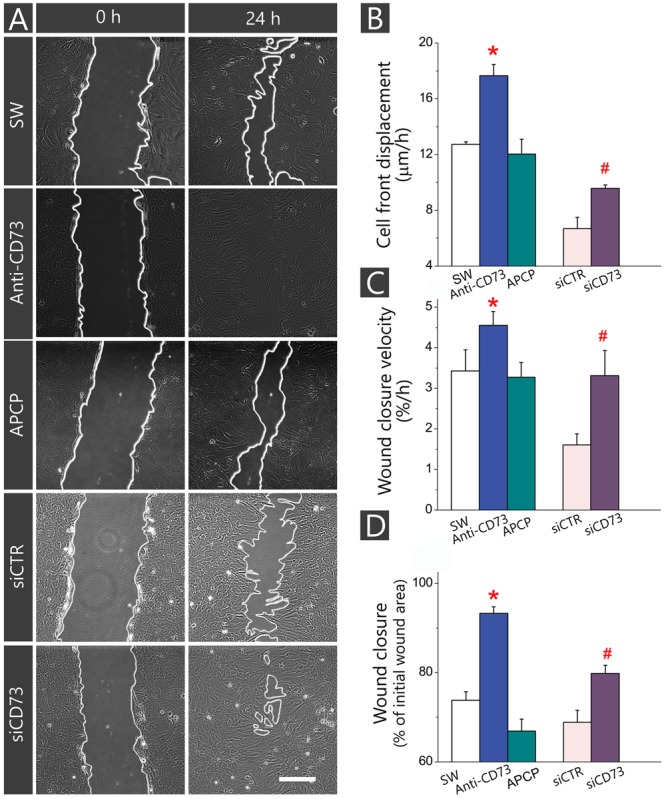
Influence of different pharmacological inhibitors of CD73 on kinetics of astrocyte migration *in vitro*. Astrocytes were grown to confluence in normal FBS and wound was made by scraping the bottom of the dish with a sterile 200-μl pipette tip. The cultures were treated with anti-CD73 antibodies and APCP and the effects on the migration were compared with non-treated SW culture. In culture transfected with siCD73, negative control of the transfection was culture transfected with non-specific siRNA duplex (siCTR). **(A)** Representative images of defined microscopic fields taken at 0 and 24 after creating the wound in cultures treated with different pharmacological inhibitors of CD73. Digitalized images were captured and analyzed in 4-h intervals during 48 h. Scale bar = 100 μm. **(B–D)** Kinetic parameters of cell migration, cell front displacement **(B)**, wound closure velocity **(C)** and wound closure **(D)** obtained in cultures treated with anti-CD73 antibodies (*blue*), APCP (*green*) or transfected with siCD73 (*magenta*). Bars represent means (±SEM) determined from 6 to 8 microscopic fields captured per each dish in *n* ≥ 7 independent culture preparations for anti-CD73 antibodies and APCP treatments and *n* = 3 for siCD73 transfection analysis. Significance inside the graphs: ^∗^ denotes significance at *p* < 0.05 in respect to SW; ^#^ denotes significance at *p* < 0.001 in respect to siCTR.

As shown in **Figure 2**, treatment with anti-CD73 antibodies increased the velocity of wound closure (**Figures 2A,B**) and promoted cell front displacement (**Figure 2C**), resulting in a complete wound area covered after 24 h (**Figures 2A–D**). Treatment with APCP, however, did not produce any apparent effect on the wound closure velocity, which was reflected in the kinetic parameters comparable to those obtained for SW. Cells with CD73 gene silencing exhibited higher velocity of a wound closure and larger wound area covered after 48 h when compared to cells transfected with non-specific siRNA (siCTR). Transfected cultures were tested in the migration assay 48 h post-transfection when the existing pool of CD73 protein and transient knock-down of CD73-mRNA were still sustained at the level of about 50%. Based on the data obtained with different pharmacological inhibitors of CD73, it was concluded that approaches that reduce expression or exposure of CD73 molecules at cell surface increase migration velocity and promote wound closure in the scratch wound assay.

### Exogenous Adenosine Does Not Affect the Motility of Astrocytes in the Scratch Wound Assay

**FIGURE 3:**
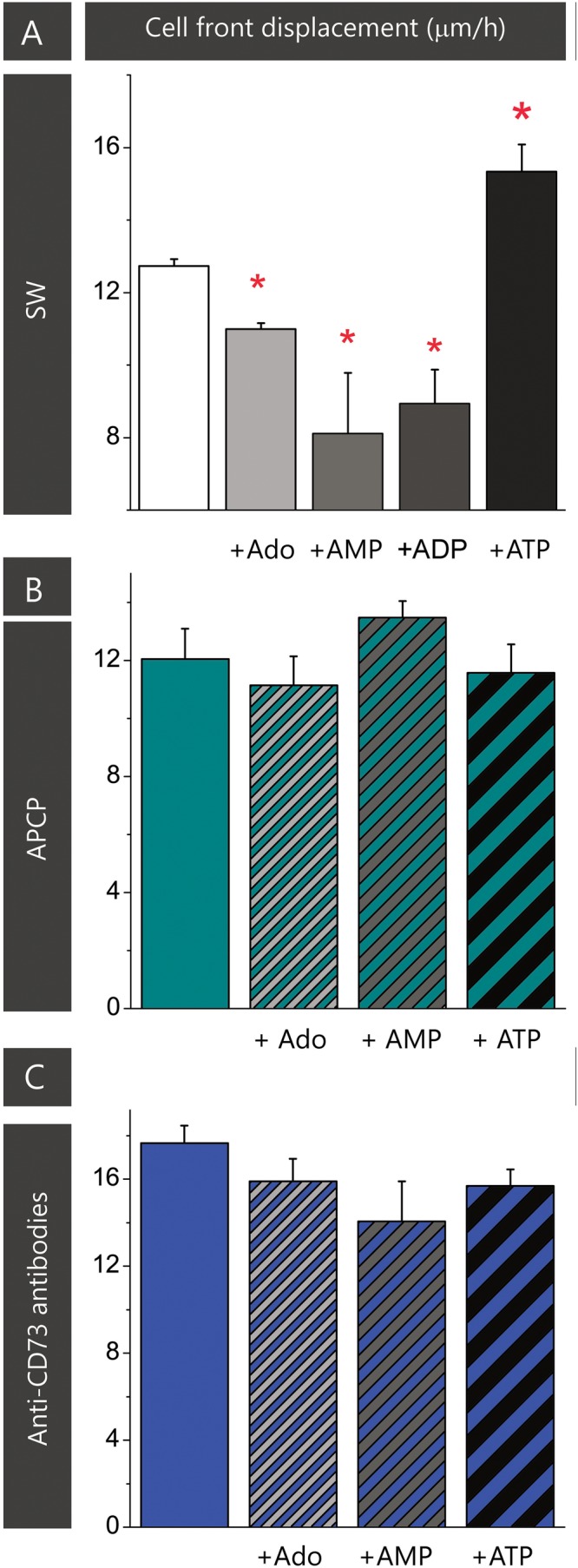
Effect of adenosine on astrocyte migration. Effect of adenosine and adenine nucleotides on astrocyte migration in SW culture **(A)**. Effect of adenosine, AMP and ATP on cell motility in the scratch wound assay in cultures treated with APCP **(B)** and anti-CD73 **(C)**. Bars represent means (±SEM) determined from 6 to 8 microscopic fields captured per each dish in *n* ≥ 7 independent culture preparations. Significance inside the graph: ^∗^ denotes significance at *p* < 0.05 in respect to SW.

The results of the previous set of scratch wound assays did not show any apparent effect of APCP on astrocyte migration. Since APCP inhibits the 5′-AMP phosphohydrolase activity of CD73, the finding further implies the lack of adenosine involvement in astrocyte migration. However, one could not exclude the possibility that APCP partially inhibits CD73, leaving the remaining activity sufficient to generate adenosine. Therefore, next set of migration assays was performed in the presence of exogenously added adenosine and short-lived nucleotides AMP, ADP, and ATP, which are efficiently converted to adenosine by ectonucleotidases action. The ability of the purine molecules to affect migration was tested in SW culture (**Figure 3A**) and in the cultures treated with the inhibitors (**Figures 3B,C**). While the addition of adenosine, AMP, and ADP notably slowed down wound closure in SW culture, the addition of ATP increased cell front displacement (**Figure 3A** and **Table 3**). When the same purine molecules were applied to cultures treated with APCP (**Figure 3B**) or anti-CD73 antibodies (**Figure 3C**), none of them induced any effect on astrocyte migration. Based on the findings presented in **Figures 2, 3**, it was concluded that although exogenous adenosine did not revert the stimulative effects of anti-CD73 antibodies and siCD73, it decreased migration velocity in SW culture, i.e., in cultures with intact CD73 function.

### The Pharmacological Inhibitors Suppress Cell Proliferation

**FIGURE 4:**
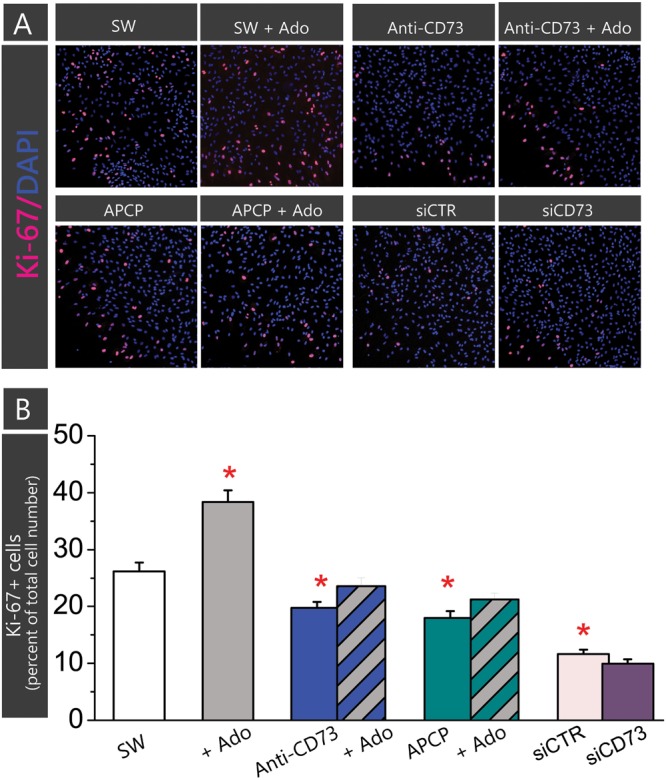
Effect of pharmacological inhibitors on cell proliferation. Cell proliferation was determined in astrocytes subjected to scratch wound and treatment with APCP or anti-CD73 in the presence or absence of exogenous adenosine, and in the culture transfected with siCD73 or siCTR and subjected to scratch wound. Cells in SW culture were kept without any treatment. Cell proliferation was determined by fluorescence labeling for Ki67^+^, which labels only dividing nuclei and DAPI^+^, which labels all cell nuclei. **(A)** Representative images of Ki-67/DAPI staining. The cultures were fixed 24 h after the treatment. **(B)** The percentage of proliferating cells in total cell number in the same field (Ki-67^+^/DAPI^+^) was counted using ImageJ. Bars present mean percentage of proliferating cells (±SEM), determined in two separate culture preparations, 5–7 frames per each treatment per multiple coverslips. Significance inside the graph: ^∗^ denotes significance at *p* < 0.05 in respect to SW.

Astrocytes in the scratch wound assay typically increase cell proliferation. To assess whether an enhanced proliferation contributes to the stimulating effect observed in cultures treated with anti-CD73 antibodies and siCD73, we next assessed cell proliferation by means of Ki-67/DAPI double fluorescence (**Figure 4**). Cells in the confluent culture proliferated at very low rate (∼2%), while cells adjacent to a scratch wound increased proliferation almost 10-fold (26.2 ± 1.5%, *p* < 0.01). In cultures treated with anti-CD73 antibodies (19.8 ± 1.0%, *p* < 0.001) and APCP (18.0 ± 1.2%, *p* < 0.001) cell proliferation decreased significantly in respect to SW, whereas in cells transfected with siCD73 cell proliferation was at the level of siCTR, but significantly lower than in non-treated control (9.9 ± 0.8%, *p* < 0.001). All applied pharmacological inhibitors exhibit anti-proliferative effect, hence they do not contribute to astrocyte migration by enhancing cell proliferation.

### Targeting CD73 With Pharmacological Inhibitors Alter the Expression Levels of Transcripts Encoding Adenosine Receptors

To enlighten an involvement of adenosine-mediated signaling in astrocyte migration, we next determined the expression levels of transcripts encoding adenosine receptors in cultures treated with the pharmacological inhibitors in the absence and presence of adenosine. As it is shown in **Table 4**, applied inhibitors distinctively affect the expression of P1 receptors, in a way that anti-CD73 antibodies significantly up-regulated the expression of A_2A_ and A_2B_ receptor subtypes and downregulated the expression of A_3_ receptor subtype. On the other hand, APCP selectively decreased the expression of A_1_R and the effect was prevented with the addition of adenosine. Adenosine alone altered the expression of A_2_R receptor subtypes.

**Table 4:** Expression levels of transcripts encoding adenosine receptors.

Target/GAPDH-mRNA abundance relative to SW (100%)					
Target	+Ado	+Anti-CD73	+Anti-CD73 + Ado	+APCP	+APCP + Ado
A_1_R	90.7 ± 11.8	117.7 ± 19.4	87.6 ± 10.6	52.4 ± 19.7^∗^	151.3 ± 10.1^#^
A_2A_R	50.7 ± 31.6^∗^	389.3 ± 20.3^∗^	286.8 ± 12.8^∗^	90.2 ± 9.1	96.4 ± 20.0
A_2B_R	147.1 ± 18.8	804.3 ± 44.4^∗^	674.9 ± 65.5^∗^	112.8 ± 27.4	139.2 ± 19.2
A_3_R	77.6 ± 36.5	21.1 ± 10.1	117.0 ± 28.4	111.2 ± 43.4	222.2 ± 67.7

*Results present mean % ± SEM, from n = 2 separate determinations performed twice in duplicate. ^∗^p < 0.05 in respect to control SW. ^#^*p* < 0.05 in respect to SW + treatment (APCP or Anti-CD73).*

### Pharmacological Inhibitors Decrease CD73 Activity, but Distinctly Affect Its Membrane Abundance in Cultured Astrocytes

**FIGURE 5:**
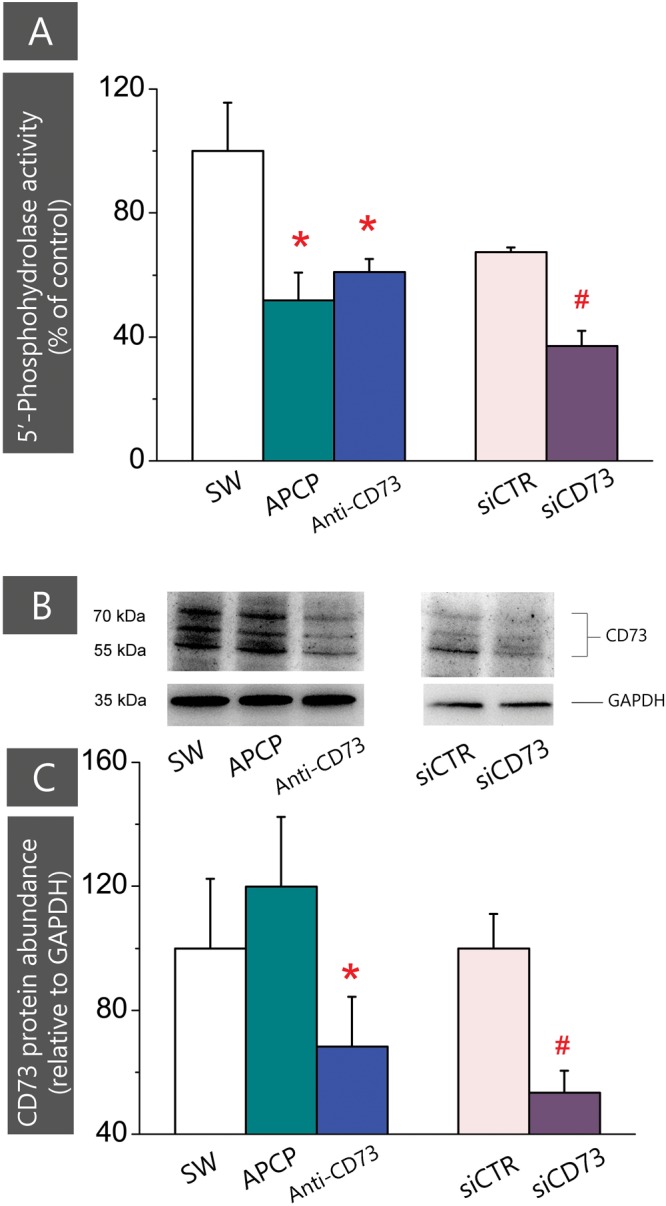
Expression of CD73 in cultures treated with different pharmacological inhibitors. **(A)** 5′-AMP phosphohydrolase activity was assayed in whole cells, after subjecting the cultures to scratch wound and treatment with APCP (*green*), anti-CD73 antibodies (*blue*) and siCD73 (*magenta*). Bars represent mean relative phospohydrolase activity (%) ± SEM, from *n* = 7 independent determinations performed in sextuplicate. ^∗^ denotes significance at *p* < 0.01 in respect to SW activity (1.13 ± 0.18 nmol Pi/mg/min); ^#^denotes significance at *p* < 0.05 in respect to siCTR activity (0.76 ± 0.02 nmol Pi/mg/min). **(B)** Representative Western blot of whole cell lysates obtained from cultures treated with different pharmacological inhibitors. Blots were probed with anti-CD73 antibodies (1:1500 in TBST; Cell Signaling, US) or anti-GAPDH antibodies. **(C)** Abundance of CD73 protein relative to GAPDH (%) ± SEM, from *n* = 3 independent determinations. CD73/GAPDH ratio obtained for SW was arbitrarily defined as 100% and used as reference. Significance level inside the graph: ^∗^ denotes significance at *p* < 0.05 in respect to SW; ^#^denotes *p* < 0.05 in respect to siCTR.

CD73 functions as phosphohydrolase which hydrolyzes 5′-AMP to adenosine. To assess to what extent applied inhibitors interfere with CD73-mediated hydrolysis, we assayed 5′-AMP phosphohydrolase in live cells treated with different inhibitors (**Figure 5A**). The activity was significantly lower in cultures treated with APCP (0.58 ± 0.11 nmol Pi/mg/min; *p* < 0.05) and anti-CD73 antibodies (0.69 ± 0.05 nmol Pi/mg/min; *p* < 0.001) than in SW (1.13 ± 0.18 nmol Pi/mg/min). Expectedly, CD73 gene silencing notably decreased 5′-AMP phosphohydrolase activity (0.42 ± 0.04 nmol Pi/mg/min; *p* < 0.001) in cells transfected with siCD73 in respect to siCTR (0.76 ± 0.02 nmol Pi/mg/min). Obtained data imply that all pharmacological inhibitors interfere with CD73 activity, yet the stimulating effect on astrocyte migration was obtained only in the cultures with a reduced surface expression of CD73.

The decrease in the enzyme activity might be due to direct catalytic inhibition of the existing enzyme molecules, or to a reduced number of enzyme molecules expressed at the cell surface. Thus, we further determined CD73 protein content in whole cell lysates by Western blotting (**Figure 5B**). As shown in **Figures 5B,C**, the abundance of CD73 protein was considerably lower in cells treated with anti-CD73 antibodies and siCD73 in respect to the corresponding controls. On the other hand, the abundance of CD73 protein in cells treated with APCP remained comparable to SW. These findings lead us to conclude that the reduced CD73 activity in cells treated with anti-CD73 antibodies was due to a lesser number of the protein molecules expressed at the cell surface, whereas the reduced CD73 activity in cells treated with APCP, was due to the catalytic inhibition of the enzyme, with no apparent influence on the enzyme protein abundance.

### Ligation of Anti-CD73 Antibodies Induces Topographical Redistribution and Decrease in the Number of CD73 Molecules at the Cell Surface

**FIGURE 6:**
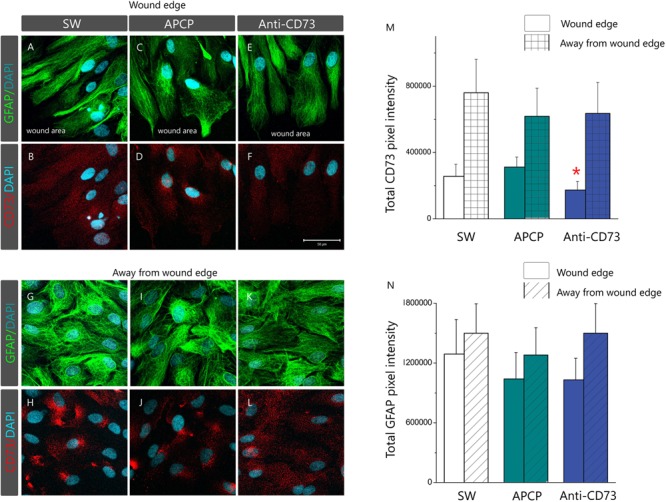
Membrane topography of CD73. Confocal images of astrocyte cultures immunostained for CD73 (*red fluorescence*) and GFAP (*green fluorescence*) and counterstained for DAPI (*blue fluorescence*). Micrographs show astrocytes in SW culture and cultures treated with APCP and anti-CD73 antibodies at the wound edge **(A–F)** and at 1–3 cell-row distance away from the wound edge **(G–L)**. Scale bar at *F* = 50 μm applicable to all micrographs. **(H)** Relative immunofluorescence intensity corresponding to CD73 **(M)** and GFAP **(N)** quantified using the ImageJ software. The results present mean integrated fluorescence density ± SEM, from two separate culture preparations and total 15 frames per treatment. ^∗^ denotes *p* < 0.05 in respect to SW.

Subcellular localization of CD73 in cultures treated with different pharmacological inhibitors was analyzed by double immunofluorescence staining for CD73 and GFAP (**Figure 6**). Based on the microscopic evaluation (**Figures 6A–L**) and distribution of total pixel immunofluorescence intensity (**Figures 6M,N**), we observed two types of astrocyte response to pharmacological inhibitors, depending on a cell distance from the wound edge. Cells lining the wound edge, irrespective of the treatment, developed protoplasmic processes extending toward the wound area (**Figures 6A,C,E**). The processes were densely labeled for GFAP, while fine punctuate CD73-*ir* was dispersed around the surface (**Figures 6B,D,F**). The overall CD73 fluorescence intensity, however, differed considerably depending on a treatment, being significantly lower in culture treated with anti-CD73 antibodies (66.2 ± 22.8%, *p* < 0.01) in respect to SW or APCP-treated culture (130.3 ± 27.1%, *p* = 0.58) (**Figure 6M**). The second type of the response was observed at 1–3 cell-row distance from the wound edge (**Figures 6G–L**). Again, irrespective of the treatment, the cells had the appearance of densely packed aggregates with strong immunoreaction for GFAP. However, in SW and APCP-treated cultures, the bulk CD73-*ir* was accumulated at few focal points at the cell surface (**Figures 6D,E**), whereas in the cells treated with anti-CD73 antibodies, the CD73-*ir* was dispersed over the cell surface (**Figure 6F**). However, the overall CD73 immunofluorescence intensities at the cells away from the wound edge were similar in all cultures. Aforementioned changes in the expression pattern of CD73 occurred without significant reorganization of the GFAP filament network or apparent changes in GFAP protein abundance (**Supplementary Figure S4**), both in the cells at the wound edge (**Figures 6A,C,E**) and in the cells away from the wound (**Figures 6G,I,K**).

### Ligation of Anti-CD73 Antibodies Induces CD73 Molecule Shedding

**FIGURE 7:**
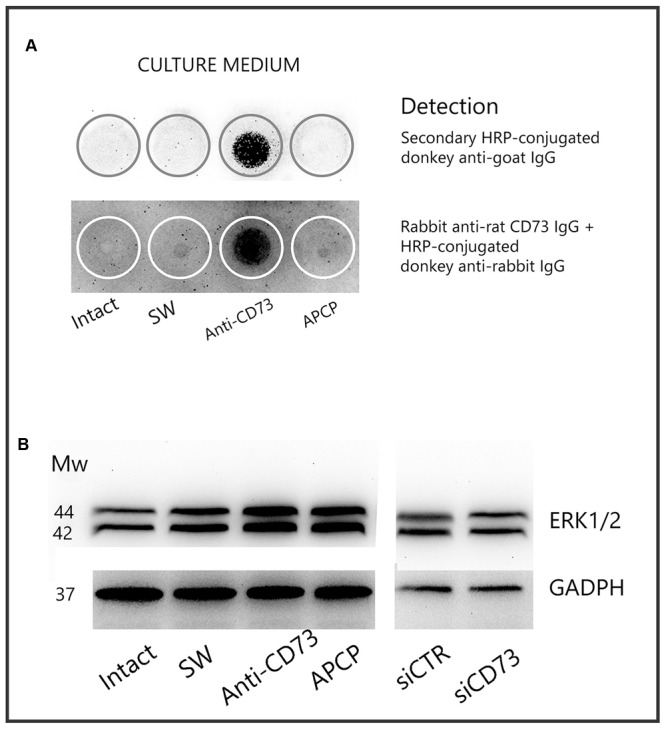
CD73 shedding. **(A)** Detection of soluble CD73 in culture media collected 24 h after the addition of the pharmacological inhibitors. Culture media were analyzed for presence of CD73 by dot blot, using either secondary IgG antibody or another set of anti-CD73 primary and matching secondary antibodies, as described in section “Materials and Methods.” Media from *n* = 4 separate cultures were used in the analysis. **(B)** Expression level of ERK1/2 in whole cell lysates obtained from cultures treated with different pharmacological inhibitors, detected by Western blotting using ERK1/2-specific antibodies.

Cleavage of a protein ectodomain is a common mechanism for ectoprotein regulation. To assess if anti-CD73 antibodies induce CD73 shedding, the presence of the soluble protein was tested in culture media, collected 24 h after the addition of the pharmacological blockades (**Figure 7A**). The culture media were first probed with secondary antibodies (donkey anti-goat IgG), matching the primary antibodies used for the pharmacological blockade (goat anti-rat CD73 IgG). Next, culture media were probed with another set of primary (rabbit anti-rat CD73 IgG; Cell Signaling) and matching secondary antibodies (Donkey anti-rabbit IgG, Invitrogen). As seen in **Figure 6A**, in both cases, soluble CD73 molecules were detected only in the media obtained from cultures treated with anti-CD73 antibodies. These findings imply that ligation of anti-CD73 antibodies with cell surface CD73 triggers a mechanism responsible for a GPI cleavage and CD73 molecule shedding.

To test whether the interaction between anti-CD73 antibodies and CD73 molecule may induce a signaling event, we examined the expression of ERK1/2 by Western blot analysis (**Figure 7B**). The expression levels of ERK1/2 increased in cells treated with anti-CD73 antibodies and APCP, whereas the levels were not altered in cells with CD73 gene silencing. These findings imply that a direct interference with CD73 molecule may trigger an intracellular signaling, further implying that CD73 participation in cell adhesion/migration depends on both the catalytic action and the adhesive properties of CD73.

## Discussion

Several experimental models of human neuropathologies studied so far demonstrate that reactive astrocytes express significantly higher level of CD73 compared to their normal counterparts (Braun et al., 1998; Bonan et al., 2000; Nedeljkovic et al., 2006, 2008; Lavrnja et al., 2009, 2015; Gandelman et al., 2010; Bjelobaba et al., 2011; Bonan, 2012). This finding is confirmed *in vitro* in the present study, by demonstrating that reactive astrocytes, irrespective of the triggering stimuli, i.e., scratch wound, LPS or IL-1β, strongly upregulate the expression and activity of CD73. However, temporal analysis of the expression *in vivo* reveals that the increase in CD73 by reactive astrocytes lags behind the increase in GFAP, and that the overall CD73 abundance peaks at fully developed reactive astrocytes already occupying the site of injury (Nedeljkovic et al., 2006; Bjelobaba et al., 2011; Lavrnja et al., 2015). While studies in human and rat glioma and medulloblastoma cell lines *in vitro* also demonstrate significantly higher levels of CD73 compared to normal astrocytes (Wink et al., 2003; Cappellari et al., 2012, 2015; Xu et al., 2013), closer analysis reveals that CD73 differentiates primary tumor cell lines, which express CD73, from metastatic cell lines which do not express the enzyme (Cappellari et al., 2012). Thus, we hypothesized that one of the roles of CD73 in astrocytes might be to act as a docking molecule which facilitates adhesion of non-migrating cells to the lamina, whereas mobile astrocytes, which move toward the site of injury, decrease the protein expression. Given the dual roles of CD73, to generate adenosine and to interact with ECM, in the present study the hypothesis has been tested by assaying migration of primary astrocytes in the presence of different pharmacological inhibitors which selectively target individual functions of CD73.

The major findings of our study are the following: (a) pharmacological inhibitors which block or reduce membrane exposure of CD73, decrease cell proliferation and increase cell migration *in vitro*, but do not affect organization of the intermediate filament network, nor decrease expression of GFAP; (b) ligation of anti-CD73 antibodies induces shedding of CD73 and the release of soluble CD73 in the culture media, and (c) the pharmacological targeting of CD73 triggers intracellular signaling events, indicating that CD73 may function as a membrane receptor which transmits activation signals into the cells. The evidence for the first notion is based on the findings that astrocytes treated with anti-CD73 antibodies or with siCD73 increase migration velocity in the scratch wound assay, while cells treated with APCP do not change migration velocity in respect to non-treated SW culture. The notion is also supported by a recent study showing that knockdown of CD73 in tumor cell lines more efficiently prevents cell adhesion to ECM than inhibition of CD73 activity with APCP (Zhi et al., 2007). Although these data together imply that adenosine has no part in astrocyte migration *in vitro*, analysis of adenosine effects in SW culture revealed that the nucleoside increased cell proliferation and reduced migration velocity in culture with functional CD73, thus indicating that CD73 and adenosine affect astrocyte migration by mutually complementing mechanisms.

It has been demonstrated that cells subjected to scratch wound increase cell proliferation (Lampugnani, 1999; Kornyei et al., 2000), whereas the cell proliferation positively correlates with CD73 expression in many different tumor cell lines, including glioma (Turnay et al., 1989; Christensen et al., 1996; Ciccarelli et al., 2000; Ohana et al., 2001; Bavaresco et al., 2008; Gao et al., 2017). Cell proliferation and CD73 expression are interconnected through TCF/LEF binding site in the regulatory region of CD73 gene, which is the nuclear target of Wnt pathway (Synnestvedt et al., 2002; Spychala and Kitajewski, 2004). This explains why factors that inhibit CD73 activity depress cell proliferation and vice versa (Andree et al., 1987; Bavaresco et al., 2008), which represent the basis for new and promising therapeutic options for cancer treatment (Antonioli et al., 2016). Our results are in agreement with the potential role of CD73 as a proliferative factor since all applied CD73 inhibitors considerably reduce cell proliferation demonstrating that the stimulatory effects of anti-CD73 antibodies and siCD73 on astrocyte migration, at least *in vitro*, cannot be attributed to increased cell proliferation.

Cell migration is a multi-step process based on a specificity of interactions and a fine balance between cell–cell and cell-substratum interactions. Involvement of several families of CAM and ECM in cell migration and adhesion is well described (Ridley et al., 2003; Wang et al., 2008), while the role of CD73 is less clear and more contradictory (Zhi et al., 2007; Wang et al., 2008; Andrade et al., 2011). Studies in normal and neoplastic cell lines have demonstrated that CD73 specifically binds tenascin C, laminin, and fibronectin (Stochaj et al., 1990; Sadej et al., 2008), with different outcomes regarding its catalytic activity and cell adhesiveness. While tenascin C strongly inhibits CD73 catalytic activity and promotes cell migration (Sadej et al., 2008), fibronectin and laminin strengthen cell adhesion, while either increasing or not affecting the activity (Olmo et al., 1992; Mehul et al., 1993). Tenascin C triggers intracellular signaling pathways, including Rho-mediated, Wnt, and FAK signaling, the latter being responsible for disruption of cell adhesion to fibronectin (Brosicke and Faissner, 2015). Thus, data suggest the possibility of CD73 playing a role in interactions between activated cells and ECM, whereas the interaction with a particular ligand determines whether the cell will adhere, migrate or completely detach. Cells actively produce ECM components and create their own extracellular environment, which in turn critically determine the cell behavior (Geiger et al., 2009). Thus, in physiological conditions, the content of tenascin C in adult brain tissue is very low and one can speculate that cell adhesion and domain organization of quiescent astrocytes are strengthened by interactions between CD73 and fibronectin. In conditions of brain injury *in vivo*, reactive astrocytes deposit large amounts of tenascin C (Wiese et al., 2012; Brosicke and Faissner, 2015) and actively change their microenvironment, which becomes more permissive for astrocyte migration, at least in part, through the altered interactions between CD73 and ECM.

The second major finding of our study is that the binding of anti-CD73 antibodies triggers CD73 molecules to shed from astrocyte membrane. Several recent studies demonstrating the potential of anti-CD73 antibodies in immunomodulation and cancer treatment, also show that specific anti-CD73 antibodies trigger CD73 to internalize (Terp et al., 2013), to shed from the membrane (this study, Airas et al., 2000; Geoghegan et al., 2016) or to remain intact (Airas et al., 1997; Sadej et al., 2006; Sadej and Skladanowski, 2012; Terp et al., 2013; Geoghegan et al., 2016), indicating that the effect may be epitope specific. Thus, it is possible that binding of anti-CD73 antibodies, depending on the exact epitope, mimics the engagement of CD73 with its natural ligands to induce CD73 shedding. Upon ligand binding, CD73 may interact with other membrane proteins, cytoskeleton components and intracellular pathways to promote stronger adhesion or migration due to internalization or shedding (Sadej et al., 2006; Terp et al., 2013). The physiological relevance of this interaction is reflected in the evidence that the interference with the CD73 molecules, by APCP or by anti-CD73 antibodies, increased the expression level of ERK1/2 in our cell culture. In the conditions *in vivo*, this scenario may be much more complex and the functional links between CD73, ECM, and intracellular signaling events have yet to be established.

Beyond these considerations, several studies demonstrate functional differences of CD73 in different cells and tissues, such as discrepancy between CD73 protein level and enzyme activity (Christensen et al., 1996; Airas et al., 1997; Cunha, 2001; Nedeljkovic et al., 2006; Stanojević et al., 2011; Brisevac et al., 2012; Lavrnja et al., 2015) or susceptibility of CD73 to ECM and lectins (Olmo et al., 1992; Mehul et al., 1993; Airas et al., 1997; Navarro et al., 1998). Data regarding the involvement of CD73 in tumor growth, invasiveness and metastasis in different tumor cell lines are even more contradictory (Antonioli et al., 2016). Since CD73 has no structural isoforms (Zimmermann, 1992), these functional variations are exhibited by CD73 molecules which are identical at protein, mRNA and cDNA level (Airas et al., 1995) but differ in their carbohydrate content. Based on the carbohydrate content, CD73 may be classified as a high-mannose type, complex carbohydrates type and hybrid type, which contains both complex carbohydrates and sialic acid residues (Meflah et al., 1984; van den Bosch et al., 1986, 1988; Wada et al., 1986; Baron and Luzio, 1987). As it is quite clear that the differences in glycosylation may be responsible for the variations found in apparent molecular weight of CD73 isolated from different sources (Turnay et al., 1989; Olmo et al., 1992; Zimmermann, 1992; Navarro et al., 1998; Grkovic et al., 2014; Lavrnja et al., 2015), it has been increasingly evident that the variations in glycan content may be responsible for the functional differences of CD73 found in different cell types and tissues. Since glycans are charged molecules, changes in the carbohydrate composition of CD73 or incorporation of negatively charged sialic acid molecules (Meflah et al., 1984), may significantly affect membrane charge density and thus cell behavior. Indeed, some earlier and recent studies demonstrate that the post-translational modifications of CD73 and alterations in the carbohydrate content may be responsible for a short-term (Vogel et al., 1991; Schoen and Kreutzberg, 1994; Lavrnja et al., 2015) and long term (Grkovic et al., 2014) regulation of CD73 functions, including its role in cell adhesion and migration. All these data point out that the regulation of CD73 by cells is a very complex mechanism, and include transcriptional and post-translational modification, together with cell-specific and tissue-specific regulators that have yet to be established.

Taken together, our results demonstrate that CD73 participates in process of astrocyte adhesion and migration, whereas both the interaction of CD73 with select ECM and the generation of adenosine may affect underlying mechanism involved in migration and adhesion. In our study, all applied pharmacological inhibitors affected certain aspect of astrocyte behavior *in vitro*; inhibition of CD73 catalytic activity did not directly affect the kinetics of a wound closure, but it decreased cell proliferation and altered the expression level of the A_1_R receptor, both effects being antagonized by the addition of adenosine. Ligation of anti-CD73 antibodies inhibited CD73 catalytic activity, decreased cell proliferation, increased migration velocity and significantly up-regulated the expression of the A_2A_R and A_2B_R of astrocytes *in vitro*, all effects being induced by CD73 molecules shedding. Both approaches altered the expression level of ERK1/2, implying that CD73 may act as a membrane receptor for the extracellular signals transmitting through interactions with ECM. Given the fact that reactive astrocytes, as well as glioma cells are highly migratory cells which express significantly higher levels of CD73 compared to their normal counterparts, it is of major scientific, clinical and pharmacological interest to discover how CD73 affects cells migration, and what is the underlying migration mechanism in the activated cells.

## Data Accessibility

Following tools, software and databases were used: Image analyses were conducted using *ImageJ* (http://imagej.nih.gov/ij/download.html; **RRID:SCR_003070**). Statistical analysis was performed using Origin 8.0 Software package (http://www.originlab.com/index.aspx?go=PRODUCTS/Origin; **RRID:SCR_014212)**.

## Author Contributions

Animal handling, cell culture preparation, treatments, rt-PCR, enzyme assay, Western blot, dot blot, immunocytochemistry and confocal microscopy imaging, cell proliferation assay, scratch wound assay were peformed by MA. Morphometric analyses: NN. Data analyses and interpretation: NN. Writing of article: NN and MA. Figure preparation: NN. Final approval of version to be published: MA and NN. Conceived and designed: NN.

## Conflict of Interest Statement

The authors declare that the research was conducted in the absence of any commercial or financial relationships that could be construed as a potential conflict of interest.

## Supplementary Material

FIGURE S1 Concentrations of pharmacological inhibitors were chosen based on their ability to block CD73, without affecting astrocyte viability. **(A)** Cells were incubated for 24 h with APCP (50–300 μM) or anti-CD73 antibodies (1:1000 to 1:250 dilution), and 5′-AMP phosphohydrolase was assayed as described. **(B)** Optimal concentration of siCD73 for transfection analysis was determined based on the ability to induce efficient transfection and inhibition of CD73 gene expression via RNA interference. There was no significant change in cell viability within applied concentrations of the inhibitors.

FIGURE S2 Expression of CD73 in cultures treated with LPS or IL-1β. **(A)** Confocal images of astrocyte cultures immunostained for CD73 (*red fluorescence*) and GFAP (*green fluorescence*) and counterstained for DAPI (*blue fluorescence*). Micrographs show astrocytes in control intact culture and intact cultures treated with 100 ng/ml LPS or 10 ng/ml IL-1β. Scale bar = 50 μm. **(B)** Relative immunofluorescence intensity corresponding to CD73, quantified using the ImageJ software. The results present mean integrated fluorescence density ± SEM, from five microscopic frames captured from one culture preparation. ^∗^ denotes *p* < 0.05 in respect to control. **(C)** 5′-AMP phosphohydrolase activity assayed in whole cells, after treating the cultures with 100 ng/ml LPS or 10 ng/ml IL-1β for 24 h Bars represent mean phosphohydrolase activity (nmol Pi/mg/min) ± SEM, from *n* = 3 determinations performed in triplicate. ^∗^ denotes significance at *p* < 0.05 in respect to control.

FIGURE S3 Kinetics of wound closure in a primary astrocyte culture. Astrocytes were grown to confluence in normal FBS and wound was made by scraping the bottom of the dish with a sterile 200-μl pipette tip. **(A)** Representative images of defined microscopic fields taken at 0–48 h after creating the wound. Scale bar = 200 μm. **(B)** Digitalized images were analyzed in ImageJ and the values of % covered area (in respect to initial wound area) at each time point were plotted vs. time to generate a growth curve. **(C)** The wound area (% of initial wound area) covered between two consecutive time-points was used to calculate the velocity of wound closure (%/h).

FIGURE S4 Representative Western blot of whole cell lysates obtained from cultures treated with different pharmacological inhibitors. Blots were probed with anti-GFAP antibodies (1:10000 in TBST) and visualized with the use of ECL solution on a Chemi Doc-It imaging system.

TABLE S1 The scratch wound assay, although very powerful to investigate cell dynamics, suffers from several disadvantages, as you pointed in your next comment. Being aware of the limitation, we performed scratching according to different geometrical patterns in distinct culture dishes, to ensure more favorable ratio between activated and non-affected cells. The number of scratches per culture dish depended on a dish diameter and type of measurements. In general, for fluorescence microscopy and visualization procedures, three scratches surrounded by several-cell wide area of intact cells were applied, whereas for the expression analysis, 5–8 scratches per dish were applied, according to the following table.
