# Supplementary figures and images for: Unveiling the Role of Ecto-5′-Nucleotidase/CD73 in Astrocyte Migration by Using Pharmacological Tools
Source: Front Pharmacol. 2018 Mar 1;9:153. doi: 10.3389/fphar.2018.00153 (PMC5837971; doi:10.3389/fphar.2018.00153)

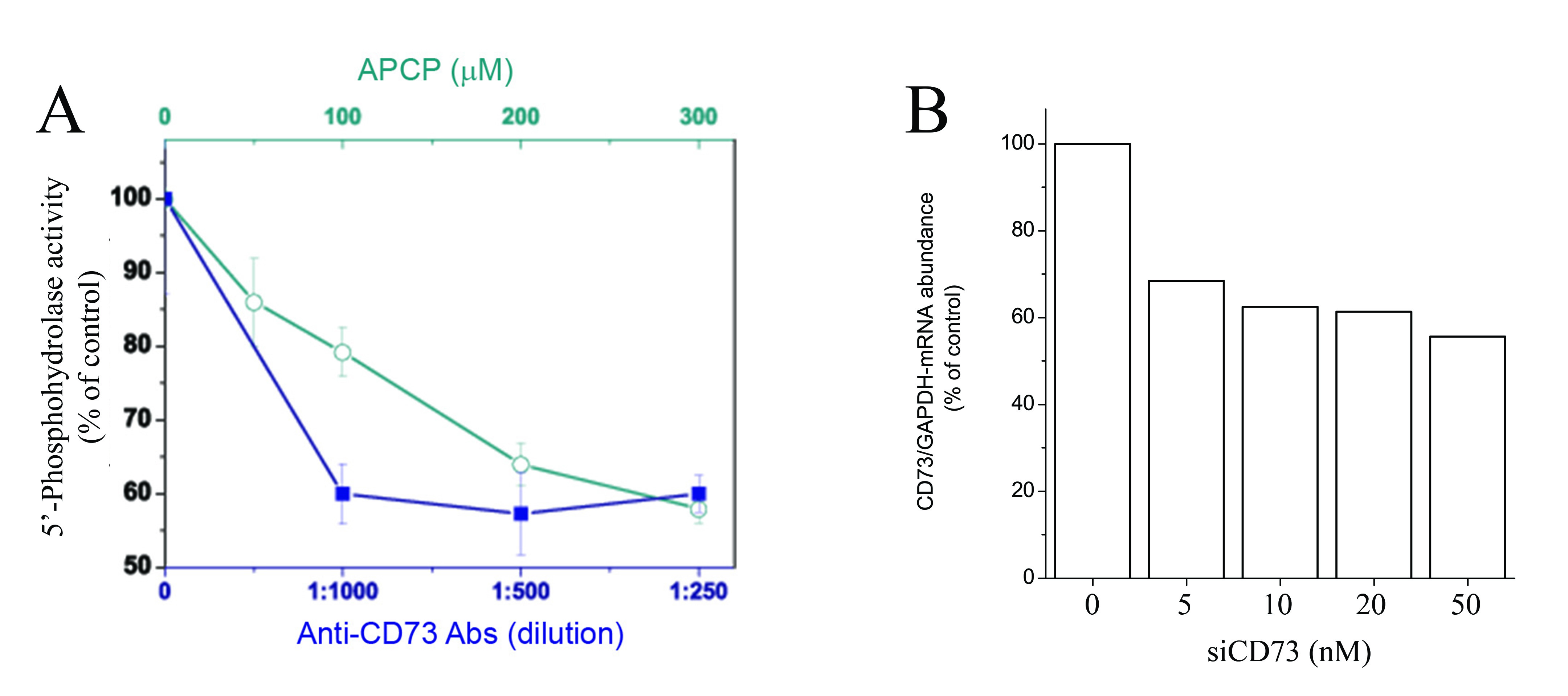

Supplement: FIGURE S1 — Concentrations of pharmacological inhibitors were chosen based on their ability to block CD73, without affecting astrocyte viability. (A) Cells were incubated for 24 h with APCP (50–300 μM) or anti-CD73 antibodies (1:1000 to 1:250 dilution), and 5′-AMP phosphohydrolase was assayed as described. (B) Optimal concentration of siCD73 for transfection analysis was determined based on the ability to induce efficient transfection and inhibition of CD73 gene expression via RNA interference. There was no significant change in cell viability within applied concentrations of the inhibitors. [file Image_1.JPEG]

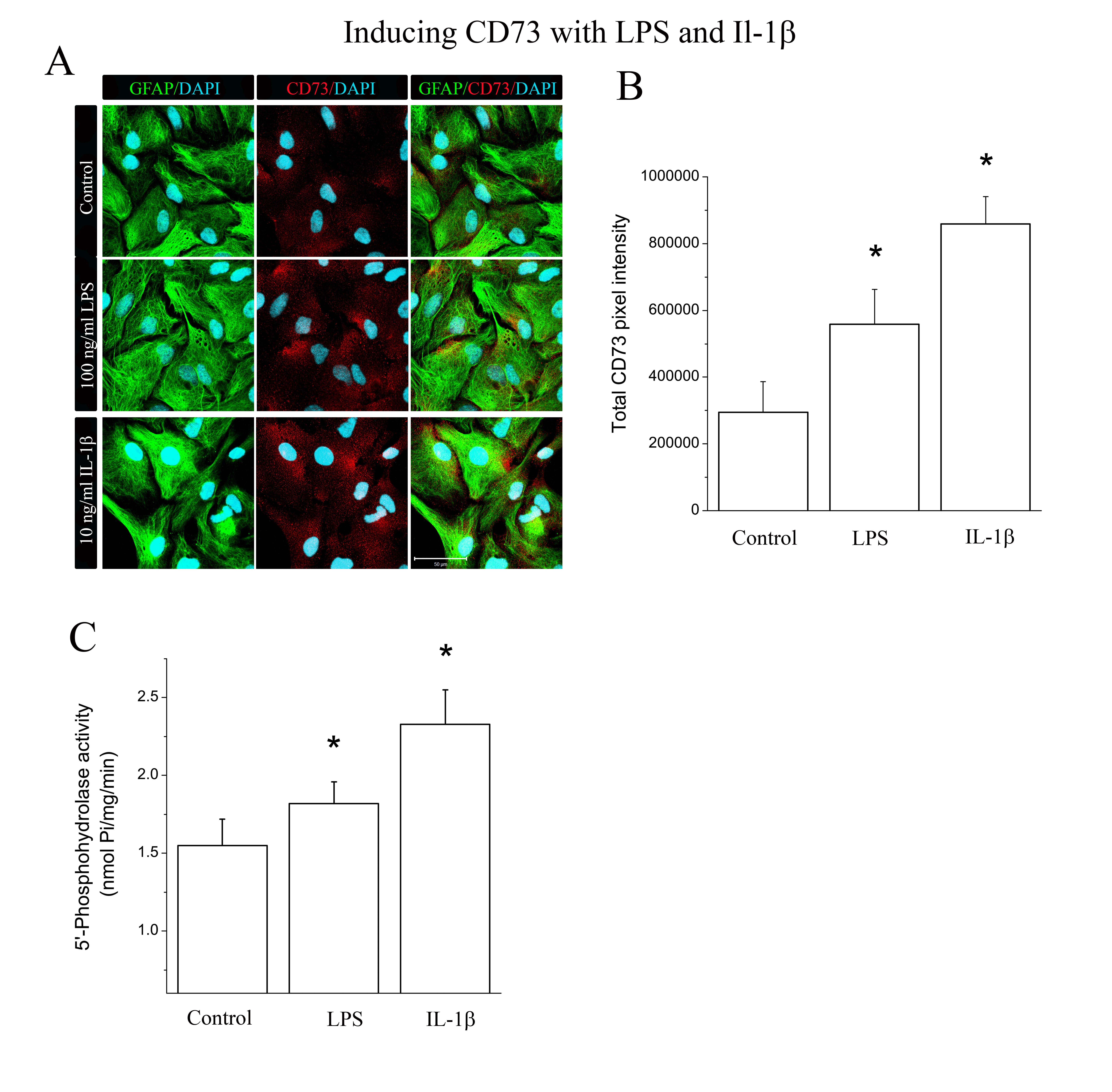

Supplement: FIGURE S2 — Expression of CD73 in cultures treated with LPS or IL-1β. (A) Confocal images of astrocyte cultures immunostained for CD73 (red fluorescence) and GFAP (green fluorescence) and counterstained for DAPI (blue fluorescence). Micrographs show astrocytes in control intact culture and intact cultures treated with 100 ng/ml LPS or 10 ng/ml IL-1β. Scale bar = 50 μm. (B) Relative immunofluorescence intensity corresponding to CD73, quantified using the ImageJ software. The results present mean integrated fluorescence density ± SEM, from five microscopic frames captured from one culture preparation. ∗ denotes p < 0.05 in respect to control. (C) 5′-AMP phosphohydrolase activity assayed in whole cells, after treating the cultures with 100 ng/ml LPS or 10 ng/ml IL-1β for 24 h Bars represent mean phosphohydrolase activity (nmol Pi/mg/min) ± SEM, from n = 3 determinations performed in triplicate. ∗ denotes significance at p < 0.05 in respect to control. [file Image_2.JPEG]

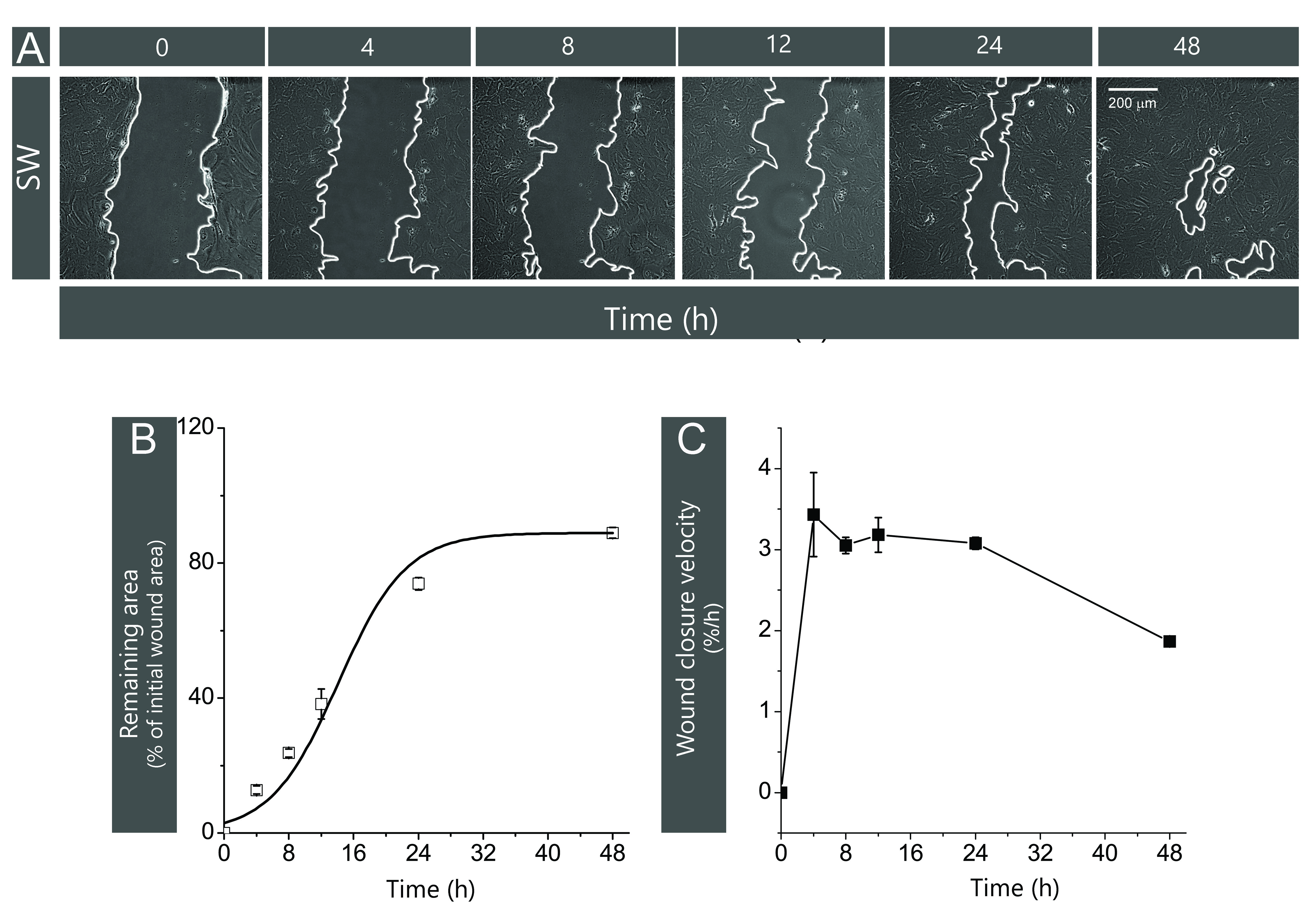

Supplement: FIGURE S3 — Kinetics of wound closure in a primary astrocyte culture. Astrocytes were grown to confluence in normal FBS and wound was made by scraping the bottom of the dish with a sterile 200-μl pipette tip. (A) Representative images of defined microscopic fields taken at 0–48 h after creating the wound. Scale bar = 200 μm. (B) Digitalized images were analyzed in ImageJ and the values of % covered area (in respect to initial wound area) at each time point were plotted vs. time to generate a growth curve. (C) The wound area (% of initial wound area) covered between two consecutive time-points was used to calculate the velocity of wound closure (%/h). [file Image_3.JPEG]

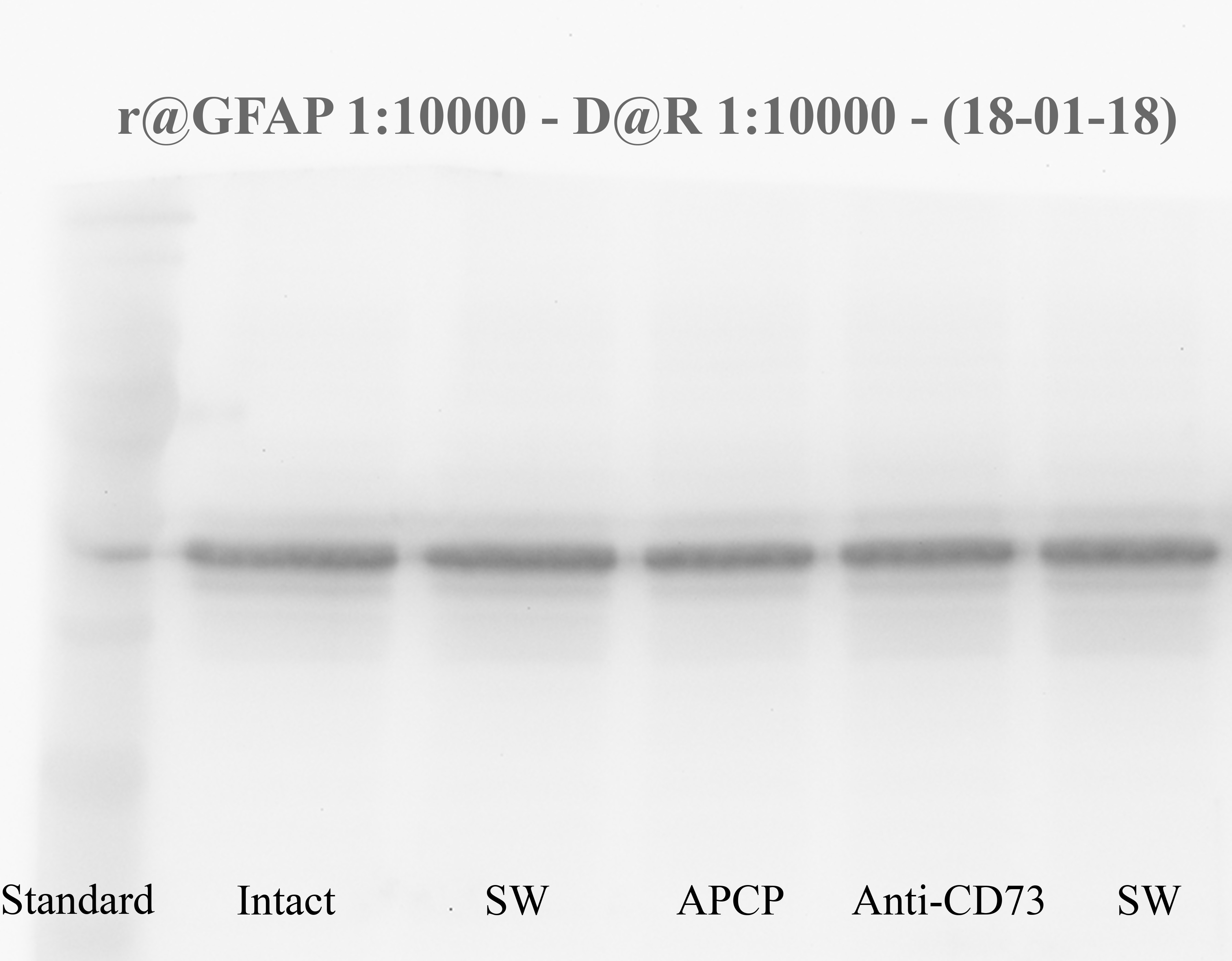

Supplement: FIGURE S4 — Representative Western blot of whole cell lysates obtained from cultures treated with different pharmacological inhibitors. Blots were probed with anti-GFAP antibodies (1:10000 in TBST) and visualized with the use of ECL solution on a Chemi Doc-It imaging system. [file Image_4.JPEG]
