# Supplementary material for: Unveiling the Role of Ecto-5′-Nucleotidase/CD73 in Astrocyte Migration by Using Pharmacological Tools
Source: Front Pharmacol. 2018 Mar 1;9:153. doi: 10.3389/fphar.2018.00153 (PMC5837971; doi:10.3389/fphar.2018.00153)
Supplement: TABLE S1 — The scratch wound assay, although very powerful to investigate cell dynamics, suffers from several disadvantages, as you pointed in your next comment. Being aware of the limitation, we performed scratching according to different geometrical patterns in distinct culture dishes, to ensure more favorable ratio between activated and non-affected cells. The number of scratches per culture dish depended on a dish diameter and type of measurements. In general, for fluorescence microscopy and visualization procedures, three scratches surrounded by several-cell wide area of intact cells were applied, whereas for the expression analysis, 5–8 scratches per dish were applied, according to the following table. [file Table_1.docx]

**Table S1.** The scratch wound assay, although very powerful to investigate cell dynamics, suffers from several disadvantages, as you pointed in your next comment. Being aware of the limitation, we performed scratching according to different geometrical patterns in distinct culture dishes, to ensure more favorable ratio between activated and non-affected cells. The number of scratches per culture dish depended on a dish diameter and type of measurements. In general, for fluorescence microscopy and visualization procedures, three scratches surrounded by several-cell wide area of intact cells were applied, whereas for the expression analysis, 5-8 scratches per dish were applied, according to the following table.

| Measurements | Culture dish | Growth area (mm^2^) | Number of scratches/dish | Wound area (% of total growth area) |
| --- | --- | --- | --- | --- |
| ICH | 15-mm cover slips | 176 | 3 | 10.2 |
| Enzyme assay | 24-well plate | 186 | 3 | 10.0 |
| SW assay,  rtPCR | 35-mm Petri dish | 924 | 5 | 7.4 |
| WB | 60-mm Petri dish | 2180 | 8 | 7.7 |
